# Early Haemodynamic Performance and Clinical Outcomes of a Novel Bioprosthetic Mitral Valve

**DOI:** 10.1093/icvts/ivaf186

**Published:** 2025-08-09

**Authors:** Yuki Wada, Akira Marui, Atsushi Nagasawa, Shinichi Tsumaru, Keisuke Hakamada, Kazuya Terazono, Yuta Kitagata, Hironori Mihara, Nobuhisa Ohno

**Affiliations:** Department of Cardiovascular Surgery, Kokura Memorial Hospital, Kitakyushu 802-8555, Japan; Department of Cardiovascular Surgery, Kokura Memorial Hospital, Kitakyushu 802-8555, Japan; Department of Cardiovascular Surgery, Kokura Memorial Hospital, Kitakyushu 802-8555, Japan; Department of Cardiovascular Surgery, Kokura Memorial Hospital, Kitakyushu 802-8555, Japan; Department of Cardiovascular Surgery, Kokura Memorial Hospital, Kitakyushu 802-8555, Japan; Department of Cardiovascular Surgery, Kokura Memorial Hospital, Kitakyushu 802-8555, Japan; Department of Cardiovascular Surgery, Kokura Memorial Hospital, Kitakyushu 802-8555, Japan; Department of Cardiovascular Surgery, Kokura Memorial Hospital, Kitakyushu 802-8555, Japan; Department of Cardiovascular Surgery, Kokura Memorial Hospital, Kitakyushu 802-8555, Japan

**Keywords:** bioprosthetic valve, mitral valve replacement, MITRIS RESILIA, structural valve deterioration, valve haemodynamics

## Abstract

**Objectives:**

The MITRIS RESILIA mitral bioprosthesis is a novel pericardial valve developed to improve durability and haemodynamic performance. However, clinical data on its use in mitral valve replacement are limited. This single-center retrospective study aimed to evaluate the early haemodynamic performance and clinical outcomes of the MITRIS RESILIA valve in patients requiring mitral valve replacement.

**Methods:**

We retrospectively reviewed data from 66 patients who underwent mitral valve replacement using MITRIS RESILIA between May 2021 and May 2024. Transthoracic echocardiography was used to assess valve haemodynamic performance at discharge, 1 year, and 2 years postoperatively. The primary end-point was early valve haemodynamics, and the secondary end-points were overall survival and structural valve deterioration.

**Results:**

The mean age of the cohort was 75.0 ± 5.8 years, and 48.5% of the patients were male. The mean pressure gradient was 3.9 ± 1.4 mmHg at discharge, 4.0 ± 2.0 mmHg at 1 year, and 3.9 ± 1.3 mmHg at 2 years. The effective orifice area was 1.52 ± 0.50 cm^2^ after surgery. None of the patients experienced structural valve deterioration or significant paravalvular leak. The rate of freedom from all-cause mortality at 2 years was 93.3%.

**Conclusions:**

MITRIS RESILIA demonstrated stable early haemodynamic performance across all valve sizes, including the 23 mm prosthesis. No cases of structural valve deterioration or paravalvular leak were observed, supporting its feasibility for mitral valve replacement. Further studies with extended follow-up are required to confirm the long-term durability and clinical benefits of the valve.

## Graphical abstract

**Figure:**
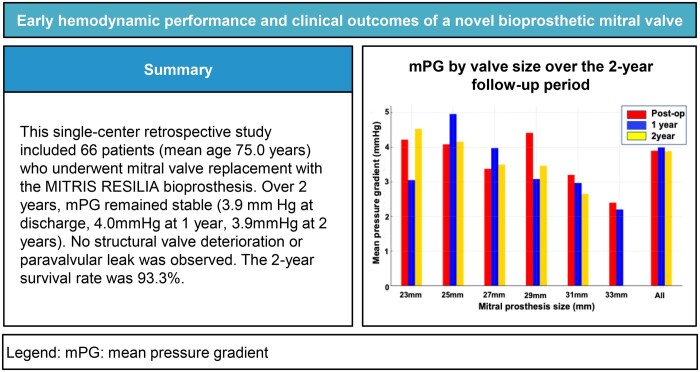

## INTRODUCTION

Mitral valve replacement (MVR) using bioprosthetic valves is a widely accepted surgical intervention for patients with mitral valve disease in whom valve repair is not feasible. However, structural valve deterioration (SVD), including calcification, remains a major limitation to the durability of bioprosthetic valves. Most commercially available bioprosthetic valves are treated using glutaraldehyde fixation to cross-link tissues and enhance structural integrity, thereby preventing early post-implantation degeneration. However, glutaraldehyde treatment is associated with *in vivo* tissue calcification, potentially leading to premature valve dysfunction.^1^ Additionally, severe calcification of glutaraldehyde-treated autologous pericardium has been reported in mitral valve repair (MVr) cases, raising concerns regarding its long-term performance and durability.^2^

The MITRIS RESILIA (Edwards Lifesciences, Irvine, CA, USA) mitral bioprosthesis, introduced as an extension of the RESILIA tissue platform, was developed to address these limitations by incorporating advanced tissue preservation techniques. The RESILIA technology, which uses functional capping, glycerolization, and an advanced terminal sterilization method, has been shown to reduce calcification in preclinical models.^3^^,^^4^ The MITRIS RESILIA valve is based on the well-established Carpentier-Edwards PERIMOUNT mitral bioprosthesis, which has demonstrated excellent long-term durability with a low SVD rate over a 20-year follow-up period.^5^ Given this foundation, the MITRIS RESILIA valve is expected to offer enhanced durability while maintaining optimal haemodynamic performance.

Although the RESILIA tissue platform has been extensively evaluated for use in aortic bioprostheses, clinical and haemodynamic data on the MITRIS RESILIA valve in the mitral position remain limited. The COMMENCE trial, a prospective, non-randomized, multicentre study evaluating RESILIA-based aortic bioprostheses, reported favourable outcomes and excellent haemodynamic performance at 2-, 4-, and 7-year follow-ups, with a mean transvalvular pressure gradient of 9.4 ± 4.5 mmHg and a 99.3% freedom from SVD rate at 7 years.^6–8^ The mitral cohort in the COMMENCE trial, which investigated the performance of Model 11000 M—a bioprosthesis structurally similar to the MITRIS RESILIA valve—demonstrated a favourable profile and stable haemodynamic performance.^9^ However, equivalent clinical and haemodynamic data for MITRIS RESILIA are currently unavailable. Given the increasing adoption of this bioprosthesis for MVR, an early assessment of its clinical and haemodynamic performance is warranted to establish its role in surgical valve therapy. This study aimed to assess the early postoperative haemodynamic outcomes of the MITRIS RESILIA valve in patients requiring MVR and provide insights into its real-world applicability and potential benefits.

## METHODS

### Ethics statement

This study was conducted in accordance with the principles of the Declaration of Helsinki and was approved by the Ethics Committee of Kokura Memorial Hospital (ID: 24111901; date: 11/19/2024). Written informed consent was obtained from all patients for the publication of this report. Any collection and storage of data or biological material from research participants for multiple and indefinite use was conducted in accordance with the WMA Declaration of Taipei. The research ethics committee approved the establishment and ongoing use of such databases and biobanks.

### Patient population

We retrospectively reviewed data from 66 patients who underwent MVR with the MITRIS RESILIA bioprosthesis for severe mitral regurgitation (MR) or mitral stenosis between May 2021 and May 2024. At our institution, although MVr is generally preferred for patients with MR, MVR was performed when the durability of repair was uncertain owing to severe degenerative changes, in cases of recurrent MR after MVr, or in those with failed transcatheter edge-to-edge repair. Patients were included regardless of whether they were undergoing concurrent procedures, such as coronary artery bypass grafting, other valvular surgeries, arrhythmia correction procedures, or repair of congenital heart disease. Patients younger than 18 years were excluded from the study.

Patient follow-up was conducted using transthoracic echocardiography at baseline (postoperatively), 3-6 months, 1 year, and 2 years to assess the haemodynamic performance of the valve. Follow-up data were available for all 66 patients postoperatively, 40 patients at 1 year, and 23 patients at 2 years. The mean follow-up duration was 1.3 ± 0.90 years.

### Surgical procedures and postoperative medications

All patients underwent MVR via either median sternotomy or right mini-thoracotomy with standard cardiopulmonary bypass. The mitral valve was accessed via a right-sided left atriotomy or a superior transseptal approach. The subvalvular apparatus of the anterior and posterior leaflets was preserved if possible. Generally, the anterior leaflet was excised and its chordae were reattached to the commissure, whereas the posterior leaflet was preserved without excision. In the presence of a calcified annulus, a thickened or calcified leaflet, a small annulus, or an infected leaflet, the leaflet and subvalvular tissue were resected. The MITRIS RESILIA valve was implanted in either the intra-annular or supra-annular position at the surgeon’s discretion.

All patients who underwent MVR with a bioprosthetic valve received anticoagulation therapy for 3 months postoperatively. The anticoagulation therapy was discontinued in patients without atrial fibrillation.

### Definitions

The use of the bioprosthetic mitral valve was considered in patients with SVD based on transthoracic echocardiography findings indicating severe regurgitation (effective regurgitant orifice area > 40 mm^2^, regurgitant volume > 60 mL) and/or stenosis (mean transvalvular gradient > 8 mmHg), regardless of whether they were asymptomatic. The effective orifice area (EOA) of the mitral valve was determined using the continuity equation based on Doppler echocardiographic measurements.

### Study end-points

The primary end-point was valve haemodynamic performance, as measured using echocardiography. The secondary end-points included early and late overall mortality and the incidence of SVD.

### Statistical analysis

All statistical analyses were performed using the R statistical software (version 4.3.1; R Foundation for Statistical Computing, Vienna, Austria). Continuous variables are presented as mean ± standard deviation or median with interquartile range. Group comparisons of continuous variables were performed using the Mann-Whitney U test, whereas categorical variables were compared using Fisher’s exact test. Kaplan-Meier survival analysis and log-rank tests were used to determine survival and event-free rates. Statistical significance was set at *P* < 0.05.

## RESULTS

### Patient characteristics

The baseline characteristics of the study population are shown in **Table 1**. The mean patient age was 75.0 ± 5.8 years. Overall, 48.5% of the patients were male, and the mean body surface area (BSA) was 1.54 ± 0.17 m^2^. Chronic or paroxysmal atrial fibrillation was noted in 66.7% of the patients. Emergency or urgent surgery was performed in 9.1% of cases, and 18.2% were redo cases. Regarding mitral valve pathology, 21.2% of the patients had stenosis, 13.6% had mitral stenosis with regurgitation, and 54.5% had isolated MR. SVD (stenosis) and SVD (regurgitation) were each noted in 1 patient (1.5%), and infective endocarditis was observed in 7.6% of the patients.

**Table 1.** Patient Characteristics

Variables	*n* = 66
Age (years)	75.0 ± 5.8
Male	32 (48.5)
BSA (m^2^)	1.54 ± 0.17
Hypertension	41 (62.1)
Diabetes mellitus	19 (28.8)
Chronic kidney disease (Cre > 1.3 mg/dL)	45 (68.2)
Haemodialysis	3 (4.5)
Dislipidaemia	27 (40.9)
Chronic obstructive pulmonary disease (> moderate)	12 (18.2)
Chronic or paroxysmal artificial fibrillation	44 (66.7)
Emergent/urgent surgery	6 (9.1)
Redo	12 (18.2)
Mitral valve pathology	
Stenosis	14 (21.2)
Stenosis with regurgitation	9 (13.6)
Regurgitation	36 (54.5)
Structural valve deterioration	2 (3.0)
Infective endocarditis	5 (7.6)

Abbreviations: BSA: body surface area; Cre: creatinine.

### Operative data

The operative data of the study population are shown in **Table 2**. The distribution of implanted valve sizes was as follows: 23 mm in 13.6% of the patients, 25 mm in 39.4%, 27 mm in 19.7%, 29 mm in 16.7%, 31 mm in 7.6%, and 33 mm in 3.0%. The mean operative time was 388 ± 89 min, with a mean cross-clamp time of 150 ± 41 min. Minimally invasive cardiac surgery was performed in 4.5% of cases.

**Table 2.** Operative Data

Variables	*n* = 66
Implanted valve size	
23 mm	9 (13.6)
25 mm	26 (39.4)
27 mm	13 (19.7)
29 mm	11 (16.7)
31 mm	5 (7.6)
33 mm	2 (3.0)
Operative time (min)	388 ± 89
Cross clamp time （min)	150 ± 41
MICS	3 (4.5)
Preservation of the subvalvular apparatus	
Anterior leaflet	0 (0)
Posterior leaflet	36 (54.5)
Bileaflet	19 (28.8)
Concomitant procedure	
Aortic valve	26 (39.4)
Tricuspid valve	48 (72.7)
CABG	5 (7.6)
Arrythmia	43 (65.2)
Congenital	5 (7.6)

Abbreviations: CABG: coronary artery bypass grafting; MICS: minimally invasive cardiac surgery.

Subvalvular apparatus preservation included posterior leaflet preservation in 54.5% of cases and bileaflet preservation in 28.8%. Notably, no isolated anterior leaflet preservation procedures were performed.

The concomitant procedures included tricuspid valve surgery (72.7%), arrhythmia-related procedures (65.2%), aortic valve surgery (39.4%), coronary artery bypass grafting (7.6%), and congenital defect repair (7.6%). In all cases categorized as congenital disease, a patent foramen ovale was closed.

### Haemodynamic performance

**Figure 1.**
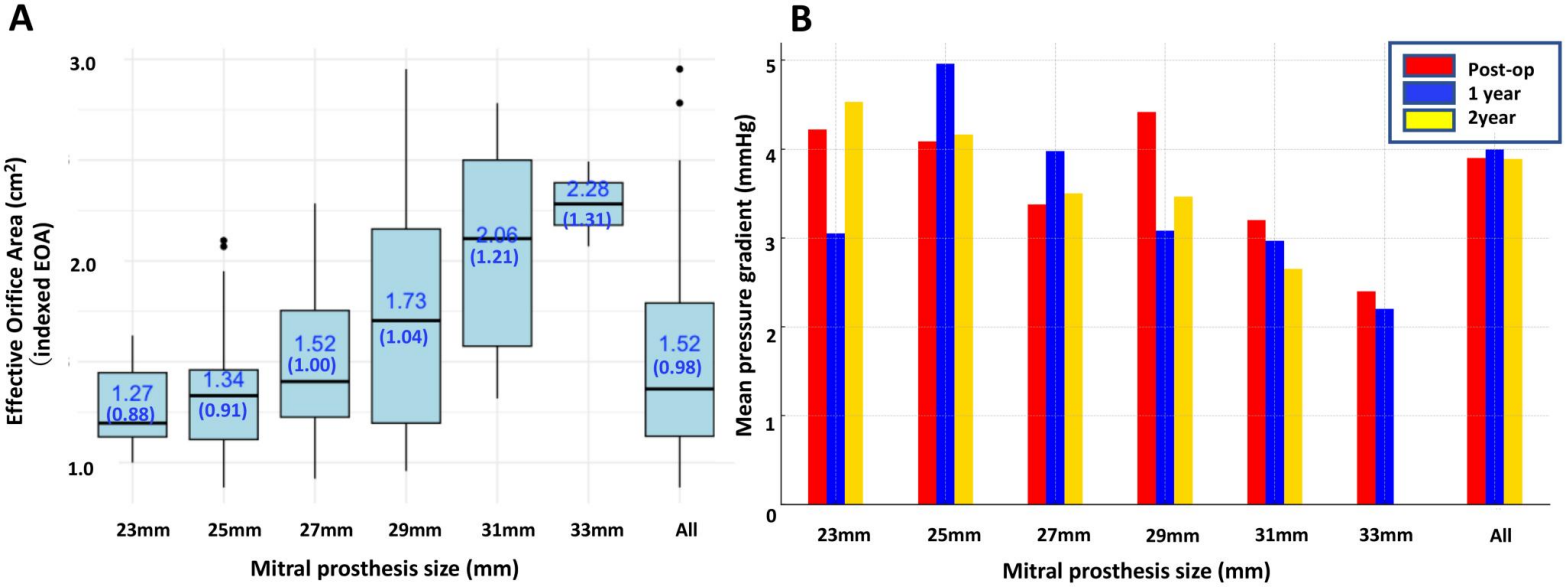
Postoperative hemodynamic performance. (A) Mean postoperative effective orifice area by valve size. (B) Mean pressure gradient by valve size over the 2-year follow-up period

The mean EOA was 1.52 ± 0.50 cm^2^, and the indexed EOA (iEOA) was 0.98 ± 0.31 cm^2^/m^2^ postoperatively. The distributions of EOA and iEOA according to valve size are shown in **Figure 1**. The mean pressure gradient (mPG) was 3.9 ± 1.4 mmHg postoperatively, 4.0 ± 2.0 mmHg at 1 year, and 3.9 ± 1.3 mmHg at 2 years for the entire cohort. The mPG by valve size at follow-up is shown in **Figure 1**. The mPG for all valves remained consistent at approximately 3-4 mmHg over the 2-year follow-up period.

No patients exhibited elevated left ventricular outflow tract (LVOT) gradients postoperatively. The mean postoperative LVOT mPG was 2.34 ± 0.92 mmHg, indicating no evidence of clinically significant LVOT obstruction.

**Figure 2.**
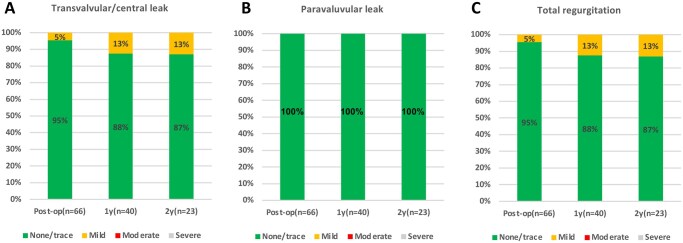
Types of valve leakage during the study period. Paravalvular (A), transvalvular (B), and total (C) leaks during the study period

Transvalvular/central, paravalvular, and total regurgitation severities are shown in **Figure 2**. The severity of transvalvular/central leak was mild in 5% of the patients postoperatively and in 13% at the 1- and 2-year follow-ups. No cases of moderate or severe regurgitation were observed, with the remaining patients exhibiting none/trivial leaks. No cases of paravalvular leak (PVL) were observed during follow-up.

### Follow-up outcomes

**Figure 3.**
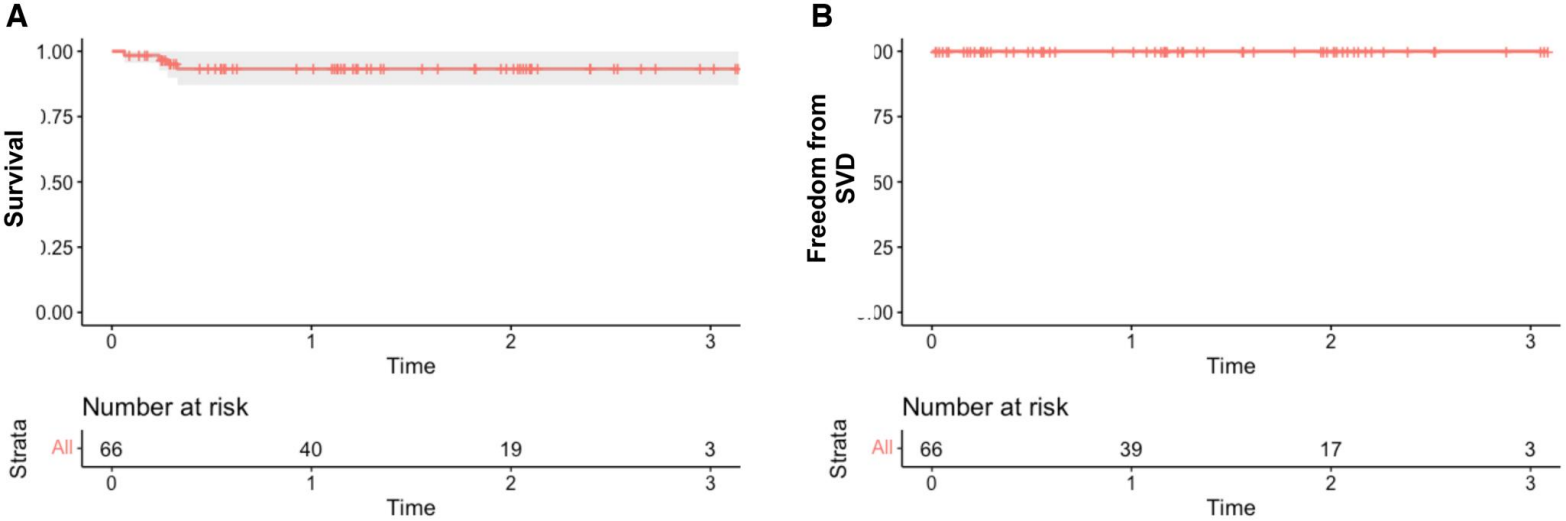
Survival and freedom from structural valve deterioration. Survival (A) and freedom from structural valve deterioration (B)

Among the 66 patients included in the study, 40 had follow-up data at 1 year and 20 at 2 years. Two cases of in-hospital mortality were reported. One patient died of intra-abdominal bleeding on postoperative day 28, whereas the other died of IgA vasculitis on postoperative day 108. Additionally, 2 patients died after hospital discharge: one owing to sepsis on postoperative day 83 and another from unknown causes on postoperative day 120. The rate of freedom from all-cause mortality at 1, 2, and 3 years was 93.3%. Furthermore, the rate of freedom from SVD at 1, 2, and 3 years was 100% (**Figure 3**). No cases of prosthetic valve endocarditis, major paravalvular leakage, or SVD were observed throughout the follow-up period.

No postoperative stroke, valve thrombosis, or transient ischaemic attack was observed during the follow-up. However, 1 case of pulmonary embolism was observed and managed appropriately.

## DISCUSSION

This study assessed the early haemodynamic performance of the MITRIS RESILIA surgical bioprosthetic mitral valve. The results showed that (1) the postoperative mPG was favourable at 3.9 mmHg and remained stable over the 2-year follow-up period; (2) the 23 mm valve demonstrated a favourable postoperative mPG of 4.2 mmHg; (3) no cases of PVL were observed during the follow-up period; and (4) no cases of SVD and prosthetic valve endocarditis occurred.

The Carpentier-Edwards PERIMOUNT mitral valves (Edwards Lifesciences, Irvine, CA, USA) have demonstrated excellent haemodynamic performance and durability.^5^ The MITRIS RESILIA surgical bioprosthetic mitral valve was developed as an evolution of the well-known and effective PERIMOUNT platform. This platform features 3 bovine pericardial leaflets, flexible stenting, and a mathematically optimized geometric design. A unique feature of this valve is the use of the RESILIA tissue for leaflet construction. The valve was commercially introduced in April 2021; however, clinical outcome data remain limited.

The mitral cohort in the COMMENCE trial, which investigated the performance of Model 11000 M—a bioprosthesis structurally similar to the MITRIS RESILIA valve—demonstrated a favourable profile and stable haemodynamic performance.^9^ However, in this trial, patients underwent MVR using a pericardial mitral bioprosthesis with RESILIA tissue. This tri-leaflet bioprosthesis is structurally identical to the Carpentier-Edwards PERIMOUNT Magna Mitral Ease valve (Model 7300TFX; Edwards Lifesciences), except that it has RESILIA tissue leaflets. Compared with the MITRIS RESILIA valve, this model has slight differences in leaflet design and fixation techniques. Additionally, the COMMENCE trial did not include a 23 mm valve, and the most frequently used valve size was 27 mm. Conversely, our study included 23 mm valves in 14% of cases, with 25 mm being the most frequently used valve size, indicating an overall use of smaller valves. In the COMMENCE trial, the mPG remained stable from discharge to 5 years (4.1 ± 2.0 mmHg at discharge and 3.7 ± 2.2 mmHg at 5 years), with predominantly trivial to mild paravalvular or transvalvular leaks observed. In our study, the mPG values were 3.9 mmHg postoperatively, 4.0 mmHg at 1 year, and 3.9 mmHg at 2 years. The severity of transvalvular/central leaks was mild in 5% of patients postoperatively and in 13% at both the 1- and 2-year follow-ups. No cases of moderate or severe leaks were noted, with all other patients exhibiting either trivial or no leaks. Importantly, no PVLs were observed during follow-up. The Epic Mitral Valve, a porcine xenograft bioprosthesis, has been reported to have a relatively high mPG of 5.5 mmHg during short-term follow-up.^10^ Furthermore, discharge echocardiographic data from other reports indicate that the 25 mm Epic valve has an mPG of 6.00 mmHg, and the 25 mm Carpentier-Edwards Perimount valve has an mPG of 5.80 mmHg.^11^ In our cohort, the 23 mm MITRIS RESILIA valve demonstrated an mPG of 4.22 mmHg, which is favourable considering the small valve size. Overall, the MITRIS RESILIA mitral valve demonstrated a favourable profile and stable haemodynamic performance in clinical use, even in patients with smaller valve sizes, such as the 23 mm valve.

Interestingly, a transient increase in the mean mitral pressure gradient was observed at 1 year, followed by a slight decrease at 2 years. This non-linear pattern has an uncertain aetiology but has been reported in previous studies, including the COMMENCE mitral cohort.^9^ Possible explanations include patient haemodynamic fluctuations, physical activity, or variability in echocardiographic measurement timing.

More than 50% of patients in our cohort received mitral valve sizes ≤25 mm. This reflects the anatomical reality of smaller annular dimensions in Asian populations, associated with lower average BSA. Rajendran et al^12^ demonstrated that mitral annular diameters in Indian adults are significantly smaller at lower BSA ranges, necessitating smaller prosthesis sizes. Similarly, Chen and Herrmann^13^ emphasized that East Asians generally have smaller annuli, particularly in the aortic position, and highlighted the clinical importance of adjusting device selection accordingly. These anatomic constraints likely explain the frequent use of smaller prostheses in our series.

In our study, the mean iEOA was relatively low (0.98 ± 0.31 cm^2^/m^2^). However, our observed EOA values were not unusually small when compared with existing literature, including those from the COMMENCE mitral cohort.^9–11^ Although some patients were classified as having prosthesis-patient mismatch (PPM) based on iEOA criteria, their mPGs remained low (approximately 4 mmHg), and no issues was observed in clinical outcomes. Regarding PPM in MVR, recent studies suggest that although mitral PPM is not uncommon, its impact on prognosis may vary considerably depending on specific clinical circumstances.^14^^,^^15^ Consequently, particularly in patients without significant comorbidities or preoperative cardiac dysfunction, numerical PPM alone may not necessarily indicate increased haemodynamic burden or poor clinical outcomes. Nevertheless, caution remains essential in prosthesis sizing, postoperative haemodynamic evaluation, and long-term monitoring to promptly identify patients potentially at risk owing to PPM.

PVL, a regurgitant flow between the prosthetic sewing ring and the valvular annulus, is a form of nonstructural valve dysfunction that can occur both early and late after prosthetic valve replacement. The reported incidence of PVL after MVR ranges from 5% to 32%.^16–20^ Annular tissue fragility in older patients and redo mitral valve surgery are identified risk factors for PVL.^16^ PVL can lead to heart failure and haemolytic anemia, significantly contributing to morbidity and mortality, particularly in a small subset of patients.^20–22^ The MITRIS RESILIA mitral valve has been reported to have improved implantability.^23^^,^^24^ Its refined saddle-shaped sewing cuff is softer than that of the PERIMOUNT Magna Mitral Ease valve, allowing better adaptation to the 3-dimensional structure of the mitral valve than conventional bioprostheses. Additionally, its low-profile design and unique flexible stent system further enhance its implantability. In this study, 12% of patients underwent redo procedures; however, no PVL of mild or greater severity was observed, indicating that the MITRIS RESILIA mitral valve offers improved implantability and annular adaptation.

The reported mortality rate associated with MVR is approximately 2%-7%.^10^^,^^16^^,^^25^ However, in the present study, the in-hospital mortality rate was approximately 3%, indicating favourable outcomes. The mitral cohort of the COMMENCE trial demonstrated very low rates of clinical events,^9^ and our findings are consistent with this trend. In the COMMENCE trial, the rate of freedom from all-cause mortality at 1, 3, and 5 years was 100%, 87.1%, and 79.9%, respectively, whereas the rate of freedom from SVD at 1, 3, and 5 years was 100%, 98.7%, and 96.7%, respectively. Our results were consistent with these findings.

We often performed concomitant tricuspid valve surgery in accordance with current international guidelines, which recommend repair in patients with moderate or greater tricuspid regurgitation or mild regurgitation with annular dilatation or persistent atrial fibrillation.

The Epic and Mosaic bioprostheses have a minimum labelled size of 25 mm, whereas the MITRIS bioprosthesis has a minimum labelled size of 23 mm. The labelled size of bioprosthetic valves is independently defined by each manufacturer, leading to inconsistencies in sizing across different brands. Studies have demonstrated that, even when valves share the same labelled size, the haemodynamic performance can vary significantly among manufacturers.^26^^,^^27^ Notably, 27 mm Epic and Mosaic bioprostheses correspond to the 25 mm MITRIS RESILIA bioprosthetic valve in terms of actual dimensions and haemodynamic performance.^26^ An *in vitro* study evaluating 8 surgical aortic valve bioprostheses with a labelled size of 21 mm revealed substantial inter-model variability in EOA and mPG.^27^ Although these findings were not based on human implantation, they emphasize the importance of considering actual valve dimensions and haemodynamic characteristics rather than relying solely on labelled sizes when selecting bioprostheses.

Additionally, the MITRIS RESILIA valve may offer structural advantages that contribute to low LVOT gradients, particularly in small patients. In a preclinical porcine model, Wang et al^26^ reported that the 25 mm MITRIS valve had the lowest stent height (7 mm) among tested mitral bioprostheses, compared to 11 mm for the Mosaic valve. This low-profile geometry may help minimize protrusion into the LVOT and reduce the risk of obstruction—an important consideration when implanting valves in patients with smaller ventricles or annular dimensions.

Although the study demonstrated favourable short-term outcomes, secondary end-points such as SVD and valve-related reinterventions generally occur after 5 to 10 years. Longer follow-up is essential to assess the long-term durability and safety of the valve.

### Limitations

This study had some limitations. First, this was a single-center retrospective study, making it susceptible to inherent limitations, including potential selection bias and confounding factors. Nevertheless, it represents the first study to date on MITRIS RESILIA prostheses implanted in a mitral position. Second, the sample size was relatively small, and echocardiographic follow-up beyond 1 year was available for only a subset of patients (40 at 1 year and 20 at 2 years), which may have limited the statistical power and generalizability of our findings. Further multicentre studies with larger cohorts are warranted to confirm these findings. Given that Japanese patients tend to have a smaller BSA, this may influence valve sizing and selection. Third, long-term follow-up data are lacking because the MITRIS RESILIA valve was only recently introduced. While SVD typically emerges beyond 5-10 years post-implantation, our study focused on short-term clinical outcomes and early haemodynamic performance. Extended follow-up is essential to assess long-term durability. Fourth, no clinical or haemodynamic data on the MITRIS RESILIA valve have been published to date, making it difficult to compare our findings with those of patients from Western countries. Evaluating the performance of the MITRIS RESILIA valve in patients with smaller body sizes is particularly important. Fifth, this is a single-arm, non-comparative study without contemporaneous Perimount Magna Ease, Mosaic, or Epic control groups. The absence of a matched comparator limits direct assessment of relative valve performance. Future multicentre studies with parallel control cohorts are warranted to validate these findings. Despite these limitations, our study provides valuable insights into the early clinical outcomes of the MITRIS RESILIA valve and establishes a foundation for future research, particularly in long-term durability assessments and comparative studies with other bioprostheses.

## CONCLUSION

In conclusion, the MITRIS RESILIA mitral bioprosthesis demonstrated favourable early postoperative haemodynamic performance in patients undergoing MVR. No cases of PVL or SVD were observed during the follow-up period. A longer follow-up period is required to confirm the sustained haemodynamic performance and safety of the valve. Future research should include direct comparisons between MITRIS RESILIA and other widely used bioprostheses, such as the Carpentier-Edwards PERIMOUNT and Epic Mitral Valve, to evaluate differences in haemodynamic function, durability, and clinical outcomes over time. Additionally, larger multicentre studies with extended follow-up will be necessary to establish its long-term safety and efficacy in diverse patient populations.

## Data Availability

The data underlying this article will be shared upon reasonable request to the corresponding author.
